# Extracellular adenosine 5ʹ-diphosphate promotes MCP-1/CCL2 expression via the P2Y_13_ purinergic receptor/ERK signaling axis in temporomandibular joint-derived mouse fibroblast-like synoviocytes

**DOI:** 10.1007/s11033-022-08125-2

**Published:** 2022-12-16

**Authors:** Seiji Yokota, Naoyuki Chosa, Shikino Matsumoto, Kazuro Satoh, Akira Ishisaki

**Affiliations:** 1 Division of Cellular Biosignal Sciences, Department of Biochemistry, Iwate Medical University, 1-1-1 Idai-dori, Yahaba-cho, Shiwa-gun, 028-3694 Iwate, Japan; 2 Division of Orthodontics, Department of Developmental Oral Health Science, Iwate Medical University, 19-1 Uchimal, 020-8505 Morioka-shi, Iwate, Japan

**Keywords:** Extracellular adenosine 5ʹ-diphosphate, Monocyte chemotactic protein 1, P2Y purinergic receptors, Extracellular signal-regulated kinase, Fibroblast-like synoviocytes, Temporomandibular joint

## Abstract

**Background:**

Temporomandibular joint osteoarthritis (TMJ-OA) causes cartilage degeneration, bone cavitation, and fibrosis of the TMJ. However, the mechanisms underlying the fibroblast-like synoviocyte (FLS)-mediated inflammatory activity in TMJ-OA remain unclear.

**Methods and results:**

Reverse transcription-quantitative polymerase chain reaction analysis revealed that the P2Y_1_, P2Y_12_, and P2Y_13_ purinergic receptor agonist adenosine 5ʹ-diphosphate (ADP) significantly induces monocyte chemotactic protein 1 (MCP-1)/ C–C motif chemokine ligand 2 (CCL2) expression in the FLS1 synovial cell line. In contrast, the uracil nucleotide UTP, which is a P2Y_2_ and P2Y_4_ agonist, has no significant effect on MCP-1/CCL2 production in FLS1 cells. In addition, the P2Y_13_ antagonist MRS 2211 considerably decreases the expression of ADP-induced MCP-1/CCL2, whereas ADP stimulation enhances extracellular signal-regulated kinase (ERK) phosphorylation. Moreover, it was found that the mitogen-activated protein kinase/ERK kinase (MEK) inhibitor U0126 reduces ADP-induced MCP-1/CCL2 expression.

**Conclusion:**

ADP enhances MCP-1/CCL2 expression in TMJ FLSs via P2Y_13_ receptors in an MEK/ERK-dependent manner, thus resulting in inflammatory cell infiltration in the TMJ. Collectively, the findings of this study contribute to a partial clarification of the signaling pathway underlying the development of inflammation in TMJ-OA and can help identify potential therapeutic targets for suppressing ADP-mediated purinergic signaling in this disease.

## Introduction

The temporomandibular joint (TMJ) comprises the mandibular condyle and fossa [1], the osteoarthritis (OA) of which is characterized by bone cavitation, cartilage deterioration, and fibrosis, ultimately resulting in TMJ pain and stiffness [2]. Previous histological studies revealed the pathogenesis of fibrosis in TMJ-OA [3, 4], indicating that the formation of fibrotic tissue can cause restricted joint movements [5]. Moreover, Zang et al. demonstrated that chronic aseptic inflammation is associated with macrophage recruitment into inflammatory lesion, which contributes to onset of fibrosis [6].

Necroptosis cell death leads to pronounced detrimental effects on tissue homeostasis by promoting the release of damage-associated molecular patterns (DAMPs), including nucleotides, high mobility group box 1, and uric acid [7]. However, endogenous ligands of purinergic receptors, such as extracellular adenine nucleotide (ATP) and adenosine 5′-diphosphate (ADP), which function as paracrine signaling molecules, have considerable potential in both pathological and physiological control of such processes [8]. Zhou et al. reported that platelet-derived ADP functions as an important mediator that promotes chondrocyte-based cartilage repair and proliferation in OA [9]. In addition, ATP and uracil nucleotide (UTP) have been shown to stimulate calcium mobilization from intercellular stores in human rheumatoid synovial cells [10].

We previously established that the fibroblast-like synoviocyte synovial cell line FLS1 (obtained from the TMJ of mouse) exhibits myofibroblastic characteristics [11]. We also demonstrated that FLS1 cells vigorously expressed the purinergic receptors P2X _3_, P2X _7_ P2Y_2_, P2Y_4_, P2Y_12_, P2Y_13_, and P2Y_14_ [12]. Typically, P2X receptors comprise ion channels that mediate the passage of cations, such as sodium, potassium, or calcium; P2Y receptors couple with the G proteins involved in the modulation of cytoplasmic Ca^2+^ concentrations and regulation of intracellular adenylyl cyclase [13]. Moreover, Jacobson et al. reported that ATP activates P2X receptors and that five or more nucleotides (including ATP, ADP, and UTP) activate P2Y receptors [14], whereas in macrophage lineage cells, extracellular ADP has also been found to promote the expression of monocyte chemotactic protein 1 (MCP-1)/C–C motif chemokine ligand 2 (CCL2) via extracellular signal-regulated kinase (ERK) signaling [15].

Chemokines play major roles in the selective recruitment of neutrophils, lymphocytes, and monocytes as well as in the activation of G protein-coupled receptors, among which MCP-1/CCL2 regulates the infiltration and migration of monocytes/macrophages [16]. However, it is yet to be established whether extracellular nucleotides affect the expression levels of MCP-1/CCL2 in FLSs.

In this study, we examined the mechanism by which DAMPs, such as ATP, ADP, and UTP, influence the expression of MCP-1/CCL2 in FLS1 cells. Specifically, we assessed whether ATP, ADP, and UTP affect the activity of mitogen-activated protein kinases (MAPKs), such as ERK, p38 MAPK, and c-Jun N-terminal kinase (JNK), in FLS1 cells. Moreover, we investigated whether ATP-, ADP-, or UTP-activated MAPKs influence the status of MCP-1/CCL2 expression in FLS1 cells. Our findings contribute to a better understanding of the signaling pathway that underlies the development of inflammation in TMJ-OA, and provide important insights regarding the potential therapeutic significance of TMJ-OA-related inflammatory activity.

## Materials and methods

### Reagents

ATP, ADP, and UTP were acquired from Merck KGaA (Darmstadt, Germany). Antagonists of P2Y_1_ (MRS 2179), P2Y_12_ (AR-C 66096), and P2Y_13_ (MRS 2211) were purchased from R&D Systems (Minneapolis, MN, USA), and U0126 and the p38 MAPK inhibitor SB203580 were purchased from Calbiochem (Merck KGaA).

### Cell culture

FLS1 cells were cultured in Ham’s F-12 medium containing 10% FBS (Invitrogen, Carlsbad, CA, USA), 2 mM glutamine, 100 U/mL penicillin, and 100 µg/mL streptomycin. To evaluate MCP-1/CCL2 mRNA expression, these cells were treated with different concentrations of ATP, ADP, and UTP for the indicated time periods. Prior to ADP administration, the cells were pre-treated for 30 min with P2Y purinergic receptor antagonists MRS 2179, AR-C 66096, and MRS 2211 to evaluate the effects of these antagonists on the ADP-induced promotion of MCP-1/CCL2 expression. In addition, prior to ADP stimulation, some cells were pre-treated for 30 min with U0126 or SB203580.

### RNA isolation and reverse transcription-quantitative polymerase chain reaction

FLS1 cells were seeded in 12-well plates (7 × 10^4^ cells/well) containing Ham’s F-12 medium for 24 h, and were thereafter cultured with or without ATP (100 µM), ADP (100 µM), or UTP (100 µM) for 24 h. Following incubation, total RNA was isolated from cells using ISOGEN reagent (Nippon Gene, Toyama, Japan). Complementary DNA was synthesized from 1 µg of the isolated RNA using a PrimeScript RT reagent kit (Takara Bio, Shiga, Japan) and used as a template for PCR performed using SYBR Premix Ex Taq II (Takara Bio) in a Thermal Cycler Dice Real Time System (Takara Bio). Amplifications were performed using the sequence-specific mouse *MCP-1* oligonucleotide primers 5ʹ-AGCAGCAGGTGTCCCAAAGA-3ʹ (forward) and 5′-GTGCTGAAGACCTTAGGGCAGA-3′ (reverse). As an internal control gene, we used mouse glyceraldehyde-3-phosphate dehydrogenase (*GAPDH*), which was amplified using the primer pair 5′-TGTGTCCGTCGTGGATCTG-3′ (forward) and 5′-TTGCTGTTGAAGTCGCAGGAG-3′ (reverse).

### Western blot analysis

FLS1 cells were seeded in 10-cm tissue culture plates at 1 × 10^6^ cells/10 cm, and thereafter cultured in serum-free Ham’s F-12 medium for 24 h. The cells were cultured with or without ADP (100 µM), with some groups being pre-treated with U0126 for the indicated times. Following incubation, total protein was extracted from the cells using RIPA lysis buffer (Sigma-Aldrich) containing protease and phosphatase inhibitor cocktails (Sigma). The protein content of each sample was measured using BCA reagent (Pierce, Rockford, IL, USA). Extracts containing equal amounts of protein were loaded on 12.5% SDS-PAGE gels, and the separated proteins were subsequently transferred to PVDF membranes and blocked with 5% skim milk in Tris-buffered saline containing Tween 20 (TBS-T) (Takara, Bio) for 1 h. The blocked membranes were then incubated overnight at 4 °C with the following primary antibodies obtained from Cell Signaling Technology, Beverly, MA, USA: ERK1/2 (#9102), phospho-ERK1/2 (T202/Y204, #9101), p38 MAPK (#9212), phospho-p38 MAPK (T180/Y182, #9211), stress-activated protein kinase (SAPK)/JNK (#9252), phospho-SAPK/JNK (T183/Y185, #9251), and GAPDH (D16H11, #5174). The following day, the membranes were incubated with appropriate secondary antibodies for 1 h, and bound antibodies were visualized using an alkaline phosphatase substrate kit (BCIP/NBT Substrate Kit; Vector Laboratories Inc., Burlingame, CA, USA). Expression of the phosphorylated target proteins was quantified using ImageJ 1.53a software (Wayne Rasband, NIH, USA).

### Statistical analysis

All experiments were performed in triplicate. The data are presented as the means ± standard deviation (n = 3). Statistical significance was evaluated using Tukey’s multiple comparison test using SPSS software (version 24.0). P-values were considered significant if *P < 0.01 and **P < 0.05.

## Results

### ADP is more effective than ATP in promoting the expression of MCP-1/CCL2 mRNA in FLSs

**Fig. 1:**
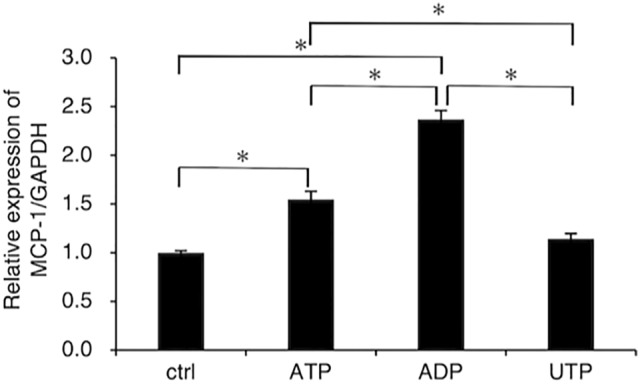
ADP promotes the expression of MCP-1/CCL2 in fibroblast-like synoviocytes. Cells were cultured with or without ATP (100 µM), ADP (100 µM), or UTP (100 µM) for 24 h. The relative expression of MCP-1/CCL2 was determined using RT-qPCR. Data are presented as the means ± standard deviation (SD) (n = 3). *P < 0.01

As shown in Fig. 1, compared to the control cells, cells treated with ATP (100 µM) and ADP (100 µM) showed 1.57-fold and 2.31-fold upregulation of MCP-1/CCL-2 mRNA expression in FLS1 cells, respectively, thereby indicating greater efficacy of ADP over ATP. In contrast, treatment with UTP (100 µM) was found to have no discernable effect on the expression of MCP-1/CCL2 mRNA in FLS1 cells.

### P2Y_13_ purinergic receptor antagonist suppresses the ADP-promoted mRNA expression of MCP-1/CCL2 in FLSs

**Fig. 2:**
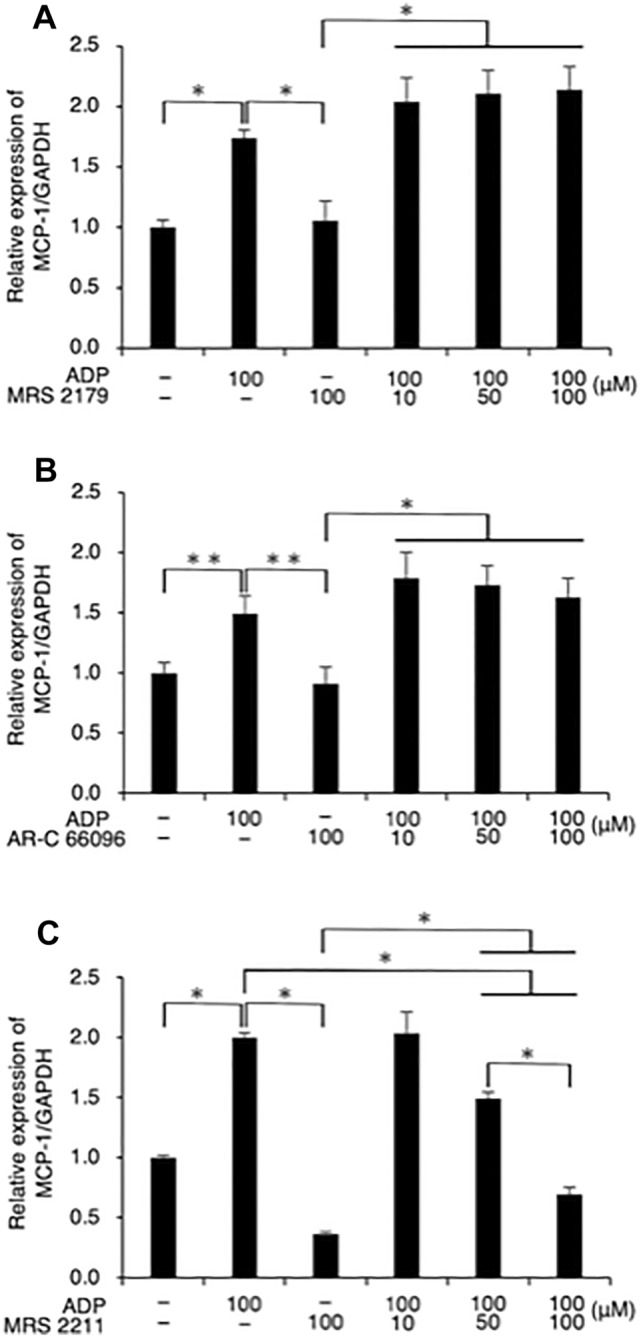
MSR 2211 concentration-dependently suppresses the expression of MCP-1/CCL2 in fibroblast-like synoviocytes. Cells were cultured with or without ADP (100 µM) for 24 h. Cells were pre-treated with the (A) MRS 2179, (B) AR-C 66096, or (C) MRS 2211 for 30 min prior to stimulation at the indicated concentrations. The relative expression of MCP-1/CCL2 was determined using RT-qPCR. Data are presented as the mean ± SD (n = 3). *P < 0.01. **P < 0.05

As shown in Fig. 2 A ,B, treatment with neither MRS2179 (10, 50, and 100 µM) nor ARC-66096 (10, 50, and 100 µM) had any appreciable effect on the ADP-promoted mRNA expression of MCP-1/CCL2 in FLS1 cells. However, MRS 2211 (50, and 100 µM) was observed to significantly reduce the ADP-promoted expression of MCP-1/CCL2 mRNA in a concentration-dependent manner (Fig. 2 C).

### ADP upregulates ERK1/ERK2 phosphorylation through P2Y_13_ receptor in FLSs

**Fig. 3:**
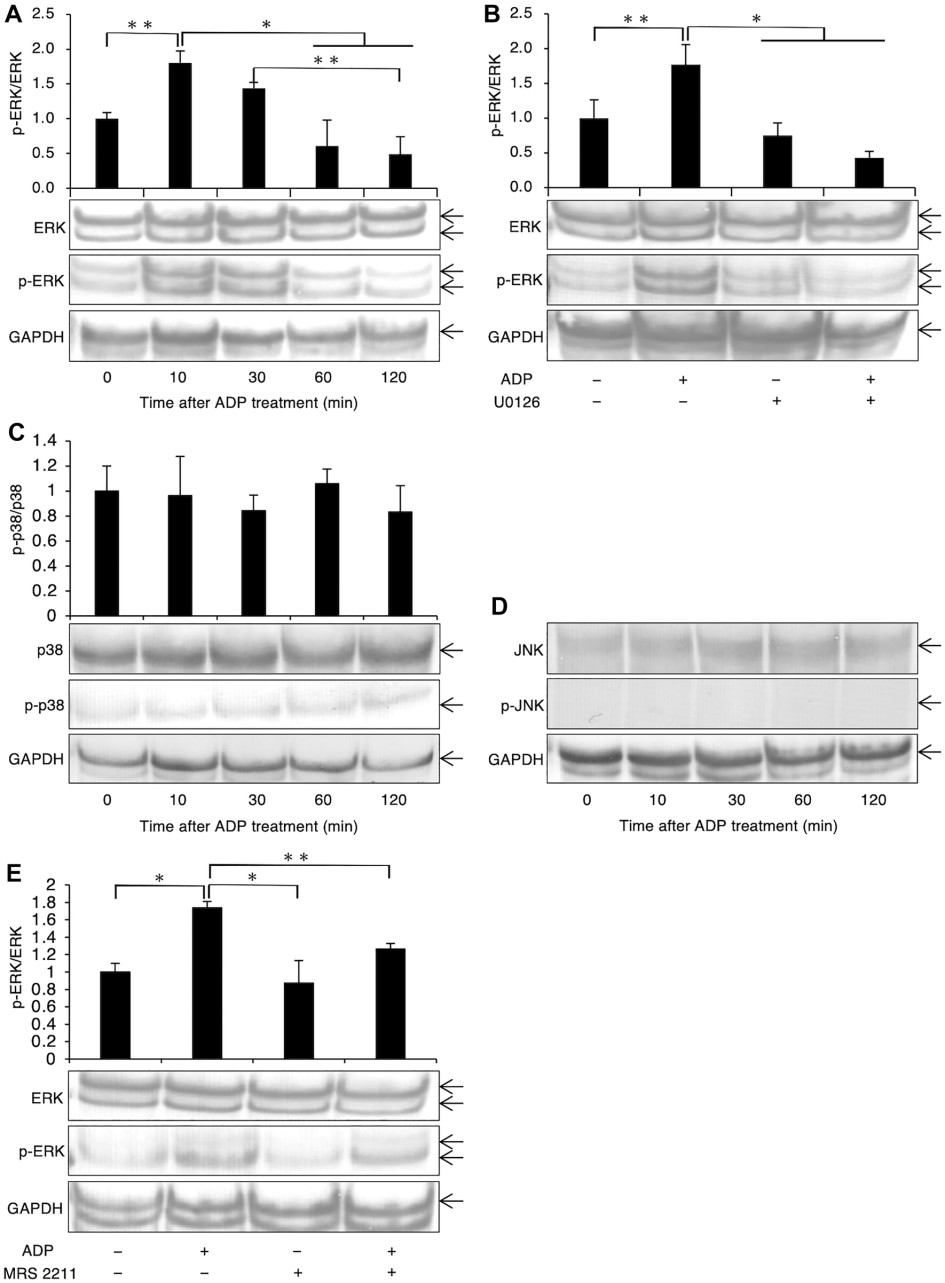
ADP upregulates ERK-1/2 phosphorylation throurh P2Y_13_ receptor in fibroblast-like synoviocytes. Cells initially were starved and then treated (**A**), (**C**), and (**D**) with ADP (100 µM) for the indicated times or (**B**), and (**E**) with or without ADP (100 µM) for 10 min. In (**B**), and (**E**), selected cell groups were pre-treated with U0126 (10 µM), or MRS 2211 (100 µM) for 30 min prior to stimulation with ADP (100 µM). ERK1/2, p38 MAPK and SAPK/JNK phosphorylation was evaluated based on western blot analysis. Data are presented as the means ± SD (n = 3). *P < 0.01. **P < 0.05

Using western blotting, we evaluated the phosphorylation status of ERK1/2 following stimulation of FLS1 cells with ADP. As shown in Fig. 3 A, ADP (100 µM) treatment led to an upregulation of ERK1/2 phosphorylation, with peak expression levels being detected 10 min after the treatment, following which, expression levels declined between 30 and 120 min post-treatment. Moreover, we established that such ADP-promoted upregulation of ERK1/2 phosphorylation could be abrogated by the administration of U0126 (10 µM) (Fig. 3B). We also confirmed that ADP (100 µM) had no significant effects on p38 MAPK phosphorylation levels (Fig. 3 C) and that SAPK/JNK remained undetectable in FLS1 cells (Fig. 3D), even after ADP stimulation. We further found that MRS 2211 (100 µM) significantly reduced the ADP-promoted upregulation of ERK1/2 phosphorylation (Fig. 3E).

### The MEK inhibitor U0126 suppresses ADP-promoted mRNA expression of MCP-1/CCL2 in FLSs

**Fig. 4:**
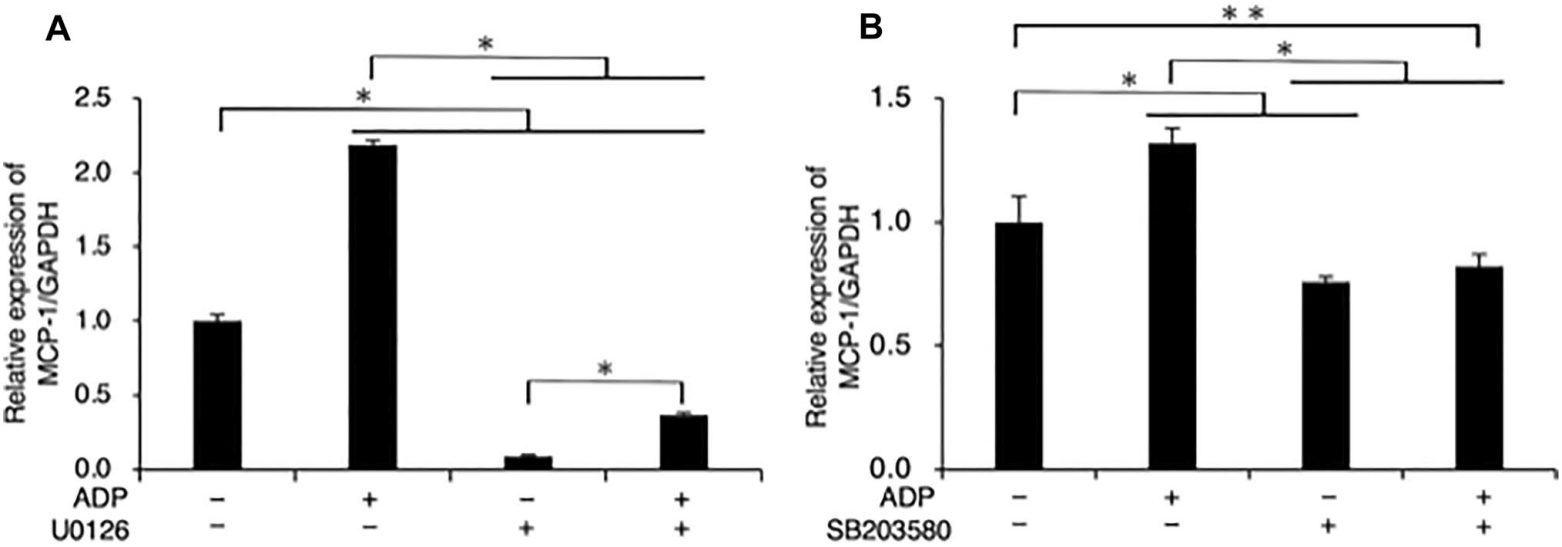
U0126, suppresses the ADP-promoted expression of MCP-1/CCL2 in fibroblast-like synoviocytes. Cells were cultured with ADP (100 µM) for 24 h. Selected cell groups were pre-treated with (**A**) U0126 (10 µM) or (**B**) SB203580 (10 µM) for 30 min prior to stimulation. The relative expression of MCP-1/CCL2 were determined using RT-qPCR. Data are presented as the means ± SD (n = 3). *P < 0.01. **P < 0.05

We also verified that U0126 (10 µM) (Fig. 4 A) and SB 203580 (10 µM) (Fig. 4B) completely abrogated the ADP-promoted expression of MCP-1/CCL2 mRNA.

## Discussion

In this study, we demonstrated that extracellularly applied ADP is more effective than ATP in promoting the expression of MCP-1/CCL2 in FLSs derived from the mouse TMJ (Fig. 1), thereby providing evidence to indicate that P2X receptor-mediated signaling might also positively regulate MCP-1/CCL2 expression in FLSs. Given that the intercellular mechanisms underlying the ATP-mediated induction of MCP-1/CCL2 expression have yet to be identified, elucidating the mechanisms associated with ATP stimulation in FLS1 cells will be the focus of our future studies. However, we established herein that UTP has no appreciable effects on MCP-1/CCL2 expression in FLS1 cells (Fig. 1). In our previous studies, we demonstrated that UTP significantly reduces mRNA expression of the fibrogenic marker α-SMA in FLS1 cells [12]. In general, UTP preferentially binds to and activates P2Y_2_, P2Y_4_, and P2Y_6_ receptors [13], and FLS1 cells strongly express P2Y_2_, P2Y_4_, P2Y_12_, P2Y_13_, and P2Y_14_ receptors [12], thereby indicating that UTP enhances the expression of α-SMA mRNA via interaction with either P2Y_2_ or P2Y_4_ receptors in these cells. Altogether, these findings indicate that UTP-induced specific intracellular signaling mediated by P2Y_2_ and P2Y_4_ receptors does not enhance the expression of MCP-1/CCL2 in FLS1 cells.

Given that ADP typically binds to P2Y_1_, P2Y_12_, and P2Y_13_, we sought to determine which subtypes of P2Y receptor mediate the promotive function of ADP in MCP-1/CCL2 expression. In this regard, we found that the P2Y_1_ antagonist MRS2179 (10–100 µM) had no effect on the ADP-promoted expression of MCP-1/CCL2 in FLSs (Fig. 2 A). Previously, Bynagari et al. demonstrated that MRS2179 (100 µM) significantly downregulates the 2MeSADP-induced phosphorylation of nPKCeta in human platelets [17], and Atterbury-Thomas et al. reported that MRS2179 (10 µM) suppresses the ADP-induced calcium increase in mouse glial cells [18]. These findings together indicate that MRS2179 concentrations in the range 10–100 µM would be optimal for antagonizing ADP-activated P2Y_1_ signaling. In addition, we also showed that the P2Y_12_ antagonist ARC-66096 (10–100 µM) had no effects on the ADP-promoted expression of MCP-1/CCL2 in FLSs (Fig. 2B). Bélanger et al. reported that ARC-66096 (10 µM) significantly inhibits platelet aggregation [19], and Quintas et al. demonstrated that ARC-66096 (10 µM) can significantly abrogate adenosine 5ʹ-*O*-(2-thio)-diphosphate-induced astroglial proliferation [20], thus indicating 10 µM to be the optimal concentration of ARC-66096 for antagonizing ADP-activated P2Y_12_ signaling. Notably, we established that the P2Y_13_ antagonist MRS2211 (50–100 µM) significantly abrogated the ADP-promoted expression of MCP-1/CCL2 in a concentration-dependent manner (Fig. 2 C). In contrast, Kim et al. demonstrated that when applied at a concentration of 30 µM, MRS2211 inhibited ADP-induced inositol triphosphate production in human astrocytoma cells [21]. In addition, Zeng et al. demonstrated that MRS2211 (100 µM) abrogated ADP-induced Ca^2+^ mobilization in primary cultured microglia [22]. Therefore, the application MRS2211 in the concentration range 30–100 µM is optimal for antagonizing ADP-activated P2Y_13_ signaling. Collectively, these findings provide convincing evidence to indicate that extracellularly applied ADP promotes the expression of MCP-1/CCL2 mRNA via the activation of P2Y_13_ receptors in FLS1 cells.

Earlier, Inose et al. demonstrated that levels of MCP-1/CCL2 mRNA expression and those of its receptor CCR2 were significantly increased in response to lysophosphatidylcholine (LPC) stimulation, and that the LPC-mediated increase in MCP-1/CCL2 transcripts was reduced by blocking the P2X receptor P2X _7_ in a microglial-derived cell line [23]. Moreover, Satonaka et al. reported that ADP upregulated the expression of MCP-1/CCL2 mRNA in cultured rat vascular smooth muscle cells (VSMCs), which was significantly inhibited by a P2Y_12_ inhibitor, thereby indicating that ADP promotes MCP-1/CCL2 mRNA expression via P2Y_12_ receptors in VSMCs [24]. Taken together, these observations imply that intracellular mechanisms underlying the extracellular nucleotide-mediated regulation of MCP-1/CCL2 expression may differ according to cell type.

In the present study, we established that ADP also promotes ERK1/2 signaling in FLS1 cells (Fig. 3 A) and confirmed that U0126, and MRS 2211 inhibits this ADP-mediated upregulation of ERK1/2 phosphorylation (Fig. 3B, and E, respectively). Moreover, U0126 partially (nevertheless significantly) abrogated the ADP-mediated upregulation of MCP-1/CCL2 mRNA expression (Fig. 4 A), thereby providing convincing evidence that ADP promotes MCP-1/CCL2 mRNA expression through P2Y_12_ receptor in FLSs in a MEK/ERK-dependent manner, which positively regulates the infiltration of monocytes/macrophages into the mouse TMJ. Zang et al. demonstrated that chronic aseptic inflammation is associated with macrophage recruitment into inflammatory lesion, which contributes to onset of fibrosis [6]. Extracellular nucleotide such as ADP derived from necrotic cells in inflammatory lesion in TMJ-OA possibly induces expression of MCP-1/CCL2 in FLSs around TMJ, which leaded to the recruitment of macrophages into the inflammatory TMJ lesion, further resulting in progression of fibrosis in TMJ-OA.

Previously, Liao et al. demonstrated that the expression of interleukin-17 (IL-17), which plays an essential role in the immune system and in the development of infectious and inflammatory diseases, upregulates MCP-1/CCL2 in RAW264.7 cells via p38 MAPK [25]. Moreover, Satonaka et al. reported that JNK/SAPK inhibition attenuates the ADP-induced upregulation of MCP-1/CCL2 mRNA and protein in VSMCs [24], whereas Ip et al. demonstrated that IL-1 and IL-13 induce MCP-1/CCL2 expression in ERK- and p38 MAPK-dependent manners in human bronchial epithelial cells [26]. Moreover, Wuyts et al. reported that IL-1β promotes the expression of MCP-1/CCL2 protein in ERK-, p38 MAPK-, and JNK/SAPK-dependent manners in human airway smooth muscle cells [27]. Collectively, these findings suggest that MAPK (ERK, p38 MAPK, and JNK/SAPK)-mediated intracellular signaling plays important roles in the expression of MCP-1/CCL2. However, in the present study, western blot analysis enabled us to confirm that ADP (100 µM) does not significantly affect the phosphorylation of p38 MAPK in FLS1 cells (Fig. 3 C), and we failed to detect JNK/SAPK, even in response ADP stimulation (Fig. 3D). These findings, therefore, provide a strong indication that at least in case of FLSs, ADP does not promote the expression of MCP-1/CCL2 via p38 MAPK or JNK/SAPK signaling pathways. We also established that the p38 inhibitor SB 203580 abrogates the ADP-promoted mRNA expression of MCP-1/CCL2 (Fig. 4B), thereby indicating that the basal activity of p38 MAPK plays an important role in the ADP-induced promotion of MCP-1/CCL2 expression in FLS1 cells.

Based on the findings of this study, we identified inflammatory molecules underlying the development of inflammation in TMJ-OA, which in turn enabled us to establish the potential therapeutic significance of TMJ-OA-related inflammatory activity. Herein, we provide convincing evidence to indicate that ADP might serve as an effective molecular target for preventing OA-related inflammation around the TMJ.

## References

[1] Ibi M (2019). Inflammation and temporomandibular joint derangement. Biol Pharm Bull.

[2] Wang XD, Zhang JN, Gan YH, Zhou YH (2015). Current understanding of pathogenesis and treatment of TMJ osteoarthritis. J Dent Res.

[3] Dijkgraaf LC, Liem RS, de Bont LG (1997). Synovial membrane involvement in osteoarthritic temporomandibular joints: a light microscopic study. Oral Surg Oral Med Oral Pathol Oral Radiol Endod.

[4] Dijkgraaf LC, Liem RS, de Bont LG (1997). Ultrastructural characteristics of the synovial membrane in osteoarthritic temporomandibular joints. J Oral Maxillifac Surg.

[5] Dij kgraaf LC, Zardeneta G, Cordewener FW, Liem RS, Schmitz JP, de Bont LG, Milam SB (2003). Crosslinking of fibrinogen and fibronectin by free radicals: a possible initial step in adhesion formation in osteoarthritis of the temporomandibular joint. J Oral Maxillifac Surg.

[6] Zang L, Xing R, Huang Z, Zang N, Zang L, Li X, Wang P (2019). Inhibition of synovial macrophage pyroptosis alleviates synovitis and fibrosis in knee osteoarthritis. Mediators Inflamm.

[7] Tang D, Kang R, Coyne CB, Zeh HJ, Lotze MT (2012). PAMPs and DAMPs: signal 0s that spur autophagy and immunity. Immunol Rev.

[8] Berenbaum F, Humbert L, Bereziat G, Thirion S (2003). Concomitant recruitment of ERK1/2 and p38 MAPK signaling pathway is required for activation of cytoplasmic phospholipase A_2_via ATP in articular chondrocytes. J Biol Chem.

[9] Zhou Q, Xu Z, Cheng X, Liu Y, Yue M, Hu M, Luo D, Niu Y, Ouyang H, Hu H (2016). Platelet promoted cartilage repair and chondrocyte proliferation via ADP in rodent model of osteoarthritis. Platelets.

[10] Loredo GA, Benton HP (1998). ATP and UTP activate calcium-mobilizing P2U-like receptors and act synergistically with interleukin-1 to stimulate prostaglandin E2 release from human rheumatoid synovial cells. Arthritis Rheum.

[11] Yokota S, Chosa N, Kyakumoto S, Kimura H, Ibi M, Kamo M, Satoh K, Ishisaki A (2017). ROCK/actin/MRTF signaling promotes the fibrogenic phenotype of fibroblast-like synoviocytes derived from the temporomandibular joint. Int J Mol Med.

[12] Matsumoto S, Yokota S, Kyakumoto S, Chosa N, Satoh K (2020) Adenosine 5ʹ-triphosphate strengthens receptor tyrosine kinase-mediated suppression of fibrogenic activity in fibroblast-like synoviocytes derived from mouse temporomandibular joints possibly through P2Y2, P2Y4, and P2Y13 purinergic receptors. Dent J of Iwate Med Univ 45:46–57

[13] Lu D, Insel PA (2014). Cellular mechanism of tissue fibrosis. 6. purinergic signaling and response in fibroblasts and tissue fibrosis. Am J Physiol Cell Physiol.

[14] Jacobson KA, Müller C (2016). Medicinal chemistry of adenosine, P2Y and P2X receptors. Neuropharmacology.

[15] Zhang X, Qin J, Zou J, Lv Z, Tan B, Shi J, Zhao Y, Ren H, Liu M, Qian M, Du B (2018). Extracellular ADP facilitates monocyte recruitment in bacterial infection via ERK signaling. Cell Mol Immunol.

[16] Deshmane SL, Kremlev S, Amini S, Sawaya BE (2009). Monocyte chemoattractant protein-1 (MCP-1): an overview. J Interferon Cytokine Res.

[17] Bynagari YS, Nagy JrB, Tuluc F, Bhavaraju K, Kim S, Vijayan KV, Kunapuli SP (2009). Mechanism of activation and functional role of protein kinase Ceta in human platelets. J Biol Chem.

[18] Atterbury-Thomas AE, Leon C, Gachet C, Forsythe ID, Evans RJ (2008). Contribution of P2Y_1_ receptors to ADP signaling in mouse spinal cord cultures. Neurosci Lett.

[19] Bélanger JC, Ferreira FLB, Welman M, Boulahya R, Tanguay JF, So DYF (2020). Lordkipainidzé: head-to-head comparison of consensus-recommended platelet function tests to assess P2Y12 inhibition—insights for multi-center trials. J Clin Med.

[20] Quintas C, Fraga S, Gonçalves J, Querios G (2011). Opposite modulation of astroglial proliferation by adenosine 5ʹ-O-(2-thio)-diphosphate and 2-methylthioadenosine-5ʹ-diphosphate: mechanisms involved. Neuroscience.

[21] Kim YC, Lee JS, Sak K, Marteau F, Mamedova L, Boeynaems JM, Jacobson KA (2005). Synthesis of pyridoxal phosphate derivatives with antagonist activity at the P2Y_13_ receptor. Biochem Pharmacol.

[22] Zeng J, Wang G, Liu X, Wang C, Tian H, Liu A, Jin H, Luo X, Chen Y (2014). P2Y13 receptor-mediated rapid increase in intracellular calcium induced by ADP in cultured dorsal spinal cord microglia. Neurochem Res.

[23] Inose Y, Kato Y, Kitagawa K, Uchiyama S, Shibata N (2015). Activated microglia in ischemic stroke penumbra upregulate MCP-1 and CCR2 expression in response to lysophosphatidylcoline derived from adjacent neurons and astrocytes. Neuropathology.

[24] Satonaka H, Nagata D, Takahashi M, Kiyosue A, Myojo M, Fujita D, Ishimitsu T, Nagano T, Nagai R, Hirata Y (2015). Involvement of P2Y_12_ receptor in vascular smooth muscle inflammatory changes via MCP-1 upregulation and monocyte adhesion. AM J Physiol Heart Circ Physiol.

[25] Liao X, Zhang W, Dai H, Jing R, Ye M, Ge W, Pei S, Pan L (2021). Neutrophil-derived IL-17 promotes ventilator-induced lung injury via p38 MAPK/MCP-1 pathway activation. Front Immunol.

[26] Ip WK, Wong CK, Lam CWK (2006). Interleukin (IL)-4 and IL-13 up-regulate monocyte chemoattractant protein-1 expression in human bronchial epithelial cells: involvement of p38 mitogen-activated protein kinase, extracellular signal-regulated kinase 1/2 and Janus kinase-2 but not c-Jun NH2-terminal kinase 1/2 signaling pathways. Clin Exp Immunol.

[27] Wuyts WA, Vanaudenaerde BM, Dupont LJ, Demedts MG, Verleden GM (2003). Involvement of p38 MAPK, JNK, p42/p44 ERK and NF-kappa B in IL-1 beta-induced chemokine release in human airway smooth muscle cells. Respir Med.

